# Effects of Vitamin D on Systemic Lupus Erythematosus Disease Activity and Autoimmunity: A Systematic Review and Meta-Analysis

**DOI:** 10.7759/cureus.25896

**Published:** 2022-06-13

**Authors:** Shayan A Irfan, Abid A Ali, Naqiha Shabbir, Hina Altaf, Ali Ahmed, Jafrikh Thamara Kunnath, Naga Vijaya L Divya Boorle, April K Miguel, Chia Chi Loh, Nikhila Gandrakota, Mirza M Ali Baig

**Affiliations:** 1 Internal Medicine, Dow University of Health Sciences, Karachi, PAK; 2 Dow Medical College, Dr. Ruth K. M. Pfau Civil Hospital Karachi, Karachi, PAK; 3 Internal Medicine, Government Medical College, Kozhikode, IND; 4 Internal Medicine, Wayne State University, Detroit, USA; 5 Internal Medicine, University of the City of Manila, Manila, PHL; 6 Internal Medicine, Manipal University College Malaysia, Melaka, MYS; 7 Family Medicine, Emory University School of Medicine, Atlanta, USA

**Keywords:** sle, anti-dsdna, sledai, vitamin d, systemic lupus erythematosus

## Abstract

This study aims to assess the role of vitamin D on systemic lupus erythematosus (SLE) patients and its effects on systemic lupus erythematosus disease activity index (SLEDAI), anti-double-stranded DNA (anti-dsDNA), C3, C4, and fatigue in patients with SLE. A systemic search was conducted using three electronic databases, i.e., PubMed/Medline, Cochrane Library, and Google Scholar. Review Manager 5.4.1 (The Cochrane Collaboration, The Nordic Cochrane Centre, Copenhagen, Denmark) was employed for statistical analysis. All studies meeting the inclusion criteria were selected. A random-effect model was used to pool the studies, and the result was reported in the standard mean difference (SMD) with its corresponding 95% confidence interval. Six randomized controlled trials were selected. Five outcomes were assessed (SLEDAI, anti-dsDNA, C3, C4, and fatigue) to evaluate the role of vitamin D in SLE patients. A significant decrease in SLEDAI (SMD = -0.85 (-1.12, -0.58); p < 0.00001; I^2^*^ ^*= 42%) and a non-significant decrease in anti-dsDNA (SMD = -0.09 (-0.03, 0.12); p = 0.42; I^2^*^ ^*= 0%) was noted. A significant increase in levels of C3 (SMD = 0.30 (0.09, 0.51); p = 0.006; I^2 ^= 0%) and fatigue (SMD = -1.27 (-2.38, -0.16); p = 0.02; I^2 ^= 56%) was noted when vitamin D was used. Insignificant difference was observed in C4 (SMD = 0.20 (-0.02, 0.41); p = 0.07; I^2^*^ ^*= 0%). Vitamin D in SLE patients showed a significant decrease in SLEDAI scores and a significant increase in C3 levels. The effect of vitamin D on fatigue was inconclusive. No significant difference in anti-dsDNA and C4 levels was noted.

## Introduction and background

Systemic lupus erythematosus (SLE) is an autoimmune multi-organ system connective tissue disorder predominantly affecting females of reproductive age, with a female to male preponderance of 9:1 [1]. The incidence varies globally with 8.6 per 100,000/year [2]. The clinical manifestation differs with joint involvement as the most common presentation along with cardiac, cutaneous, neurologic, hematologic, and renal presentations. Due to the intricate nature of this disease, different disease activity tools have been established with the systemic lupus erythematosus disease activity index (SLEDAI) as the globally accepted primitive assessment scale. This index is based upon 24 clinical and laboratory parameters ranging from scores 1 to 8 with a maximum score of 105 [3]. Different modified versions of the scale are now devised, which include SELENA-SLEDAI, SLEDAI-2K, and SLEDAI-2K (30 days) [4].

The hallmark of the disease is the production of autoantibodies (antinuclear antibodies (ANA), anti-double-stranded DNA (anti-dsDNA), and anti-Sm) due to the inability to maintain self-tolerance by the immune system [5]. The interaction of environmental factors, such as exposure to ultraviolet radiations, certain infections (Epstein-Barr virus, parvovirus B19, and retrovirus), and drugs like hydralazine and procainamide [6], with genetic mutation of human leukocyte antigen (HLA) and Fcγ receptor genes along with IRF5, STAT4, PTPN22, TNFAIP3, BLK, BANK1, TNFSF4, and ITGAM is involved in the pathogenesis via type 1 interferon and Toll-like receptor signaling pathways [7]. They damage cells that undergo apoptosis and release a pool of cytokines with interferon alpha (INF-α) and nucleic antigens that activate T cells and B cells resulting in the production of ANA and immune complex formation [8] that deposit in blood vessels, kidneys, connective tissue, and skin. Due to the extensive involvement of multiple organs, a higher level of fatigue is observed in patients with SLE [9]. The reduced level of vitamin D along with mood and anxiety disorders is responsible for fatigue in SLE [10].

The imbalance in immunomodulation is influenced by the levels of circulating vitamin D in serum. Vitamin D augments innate immune response, promotes self-tolerance, and mitigates the Th1 cell proliferation [11]. It inhibits the proliferation of activated B cells. B cells express vitamin D receptor (VDR), and the binding of calcitriol (1,25(OH)₂D₃) to VDR mediates the antiproliferative action on B cells [12]. Islam et al., in a systematic review and meta-analysis, observed significant low levels of vitamin D in SLE patients [13]. The prevalence of vitamin D deficiency in SLE ranged from 4% to 54% [14]. However, it is unclear whether low levels of vitamin D act as a risk factor for SLE or a consequence of the disease. Patients with SLE are advised to avoid sun exposure and certain medications prescribed, such as glucocorticoid, which is an immunosuppressant but reduces the absorption of vitamin D from the intestine and enhances its breakdown, further reducing the vitamin D status in the blood. Zheng et al. (2019) conducted a meta-analysis evaluating the effectiveness of vitamin D supplements on SLE patients in comparison to controls; however, the study includes a smaller number of articles and one retracted study [15]. In our literature review, no systematic review and meta-analysis evaluating the role of vitamin D before and after treatment have been published before. Therefore, we conduct this systematic review and meta-analysis based on randomized controlled trials (RCTs) to assess the effectiveness of vitamin D on SLE patients.

## Review

Method

Data Sources and Search Strategy

The systematic review and meta-analysis were conducted following the guidelines of the Preferred Reporting Items for Systematic Reviews and Meta-Analyses (PRISMA) [16]. An electronic search using Medline, Cochrane Trial Register, and Google Scholar was conducted from their inception to September 15, 2021. The following search string was used: (SLE OR systemic lupus erythematosus OR lupus OR lupus erythematosus) AND (vitamin D OR Drisdol OR Calciferol OR cholecalciferol OR 1,25-dihydroxycholecalciferol OR ergocalciferol). We additionally screened the cited articles of previously published meta-analyses, cohort studies, and review articles to identify any relevant studies.

Study Selection

Studies were only included if they met the following eligibility criteria described as PECOS: (1) population: diagnosed SLE patients; (2) exposure: vitamin D supplementation; (3) control: baseline characteristics; (4) outcome: effectiveness of vitamin D on disease activity and autoimmunity; (5) studies: RCTs.

Data Extraction

The electronic databases were screened by two reviewers. Studies were exported to EndNote Reference Library version 20.0.1 (Clarivate Analytics, London, UK) and duplicate articles were screened and removed. Two investigators independently extracted data from the selected studies on a computer spreadsheet. Any discrepancy was resolved through consensus discussions among the investigators.

Quality Assessment of Studies

The quality of each included study was assessed by two investigators independently. The Grading of Recommendations Assessment, Development, and Evaluation (GRADE) approach was used to assess the risk of bias from RCTs in seven domains: adequate sequence generation, allocation concealment, blinding of participants and personnel, blinding of outcome assessment, incomplete outcome data, selective outcome reporting, and free of other bias [17]. The individual domains and overall risk-of-bias judgment were expressed on one of three levels: high risk of bias, unclear risk of bias, and low risk of bias. Based on these factors, the overall quality of evidence was deemed as high, moderate, or low risk of bias.

Statistical Analysis

Review Manager version 5.4.1 (The Cochrane Collaboration, The Nordic Cochrane Centre, Copenhagen, Denmark) was employed for all the statistical analyses. The data from the included studies were pooled using a random-effects model. The analysis of the results was done by calculating the standard mean difference (SMD) with respective 95% confidence intervals (CI). The chi-square test was used to evaluate any discrepancy between the subgroups. The sensitivity analysis was performed to assess if any individual study was responsible for driving the results and imploring reasons for high heterogeneity. According to Higgins et al., scale for heterogeneity was considered as follows: I^2^ = 25-50% (moderate heterogeneity), 50-75% (substantial heterogeneity), and 75-100% (considerable heterogeneity). P-value < 0.1 indicated significant heterogeneity [18]. The results of the analysis were considered significant if p < 0.05.

Results

Literature Search Results

**Figure 1:**
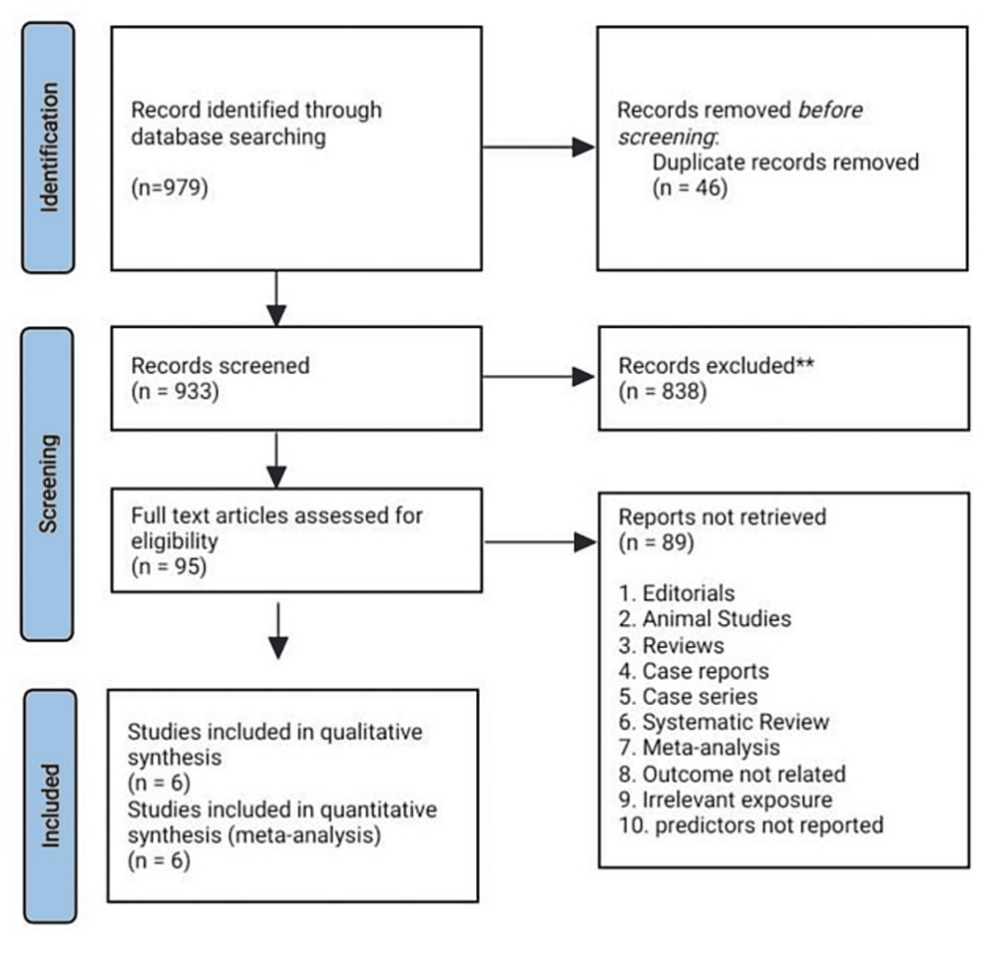
PRISMA flow diagram PRISMA: Preferred Reporting Items for Systematic Reviews and Meta-Analyses.

The initial search using three electronic databases yielded 979 potential studies. A total of 95 studies were selected after the exclusion based on the title and the abstract. Six studies remained for quantitative analysis after reading the full text. Figure 1 summarizes the results of our literature search.

Study Characteristics

Tables 1, 2 provide the demographic and clinical characteristics of the included studies [19-24]. The analysis included six published RCTs, with a patient population of 276. Two studies were from Indonesia, one from Italy, one from Iran, one from Brazil, and one from Bangkok. The average age of the patients in these studies was 30.24 years.

**Table 1:** Baseline clinical characteristics of the study population with vitamin D supplementation SR: standard regimen; IR: intensive regimen; anti-dsDNA: anti-double-stranded DNA; IL-6: interleukin 6; TGF-β1: transforming growth factor beta 1.

Study	SLEDAI	Vitamin D dosage	Other medications	Factors present	Risk of bias
Andreoli et al. [19]	2 ± 2.96	SR = 25,000 IU/month, IR = 300,000 IU bolus + 50,000 IU/month	Glucocorticoids, antimalarials, and immunosuppressive drugs	Anti-dsDNA, C3, C4	Moderate risk
Lima et al. [20]	3.0 ± 3.22	50,000 IU/week	Glucocorticoids, antimalarials, and immunosuppressive drugs	Anti-dsDNA, C3, C4, fatigue	Low risk
Rifa’i et al. [21]	12.65 ± 4.85	1200 IU/day	Non-immunosuppressants, azathioprine, cyclophosphamide, methyl mycophenolate (MMF), chloroquine	Fatigue Severity Score (FSS), anti-dsDNA	Low risk
Karimzadeh et al. [22]	3.09 ± 2.36	50,000 IU/week for 12 weeks, 50,000 IU/month for 3 months	Glucocorticoids, antimalarials, immunosuppressive drugs	Anti-dsDNA, C3, C4	Low risk
Wahono et al. [23]	15.2 ± 7.4	3 x 400 IU	Curcuma xanthorrhiza, corticosteroids, chloroquine, cyclophosphamide, mycophenolate mofetil, azathioprine, cyclosporine	Anti-dsDNA, C3, C4, IL-6, TGF-β1	Low risk
Pakchotanon et al. [24]	4 ± 2.96	100,000 IU (4 weeks), 40,000 IU (20 weeks)	Cholecalciferol (800 units), glucocorticoids, antimalarials, immunosuppressive drugs	Anti-dsDNA, C3, C4	Low risk

**Table 2:** Demographic characteristics of study participants with vitamin D supplementation

Study	Year	Duration	Country	Participants with vitamin D supplementation (IU)	Mean age (years)
Andreoli et al. [19]	2015	24 months	Italy	34	31.83
Lima et al. [20]	2016	24 weeks	Brazil	20	18.5
Rifa’i et al. [21]	2016	3 months	Indonesia	20	28.25
Karimzadeh et al. [22]	2017	3 months, 12 weeks	Iran	45	33.78
Wahono et al. [23]	2017	14 months	Indonesia	20	27.9
Pakchotanon et al. [24]	2020	24 weeks	Bangkok	52	41.15

Publication Bias and Quality Assessment

Publication bias cannot be assessed since the number of included studies was less than 10. All studies had a low risk of bias except for one study, which had a moderate risk of bias [18]. Details of the GRADE approach are provided in the Appendix.

Results of meta-analysis

Detailed forest plots outlining the effect size of SLEDAI, anti-dsDNA, C3, C4, and fatigue are provided below.

Effect of Vitamin D on SLEDAI in SLE Patients

Five studies were used to analyze the effect of vitamin D on SLEDAI in SLE patients [20-24]. Pakchotanon et al.'s study was divided into two studies based on the dosage level [24]. After intervention group had 209 patients while the before intervention group also had 209 patients. Analysis was done by subgrouping the intervention period greater than 24 weeks with mild SLEDAI scores and the intervention period less than 24 weeks with high SLEDAI scores.

**Figure 2:**
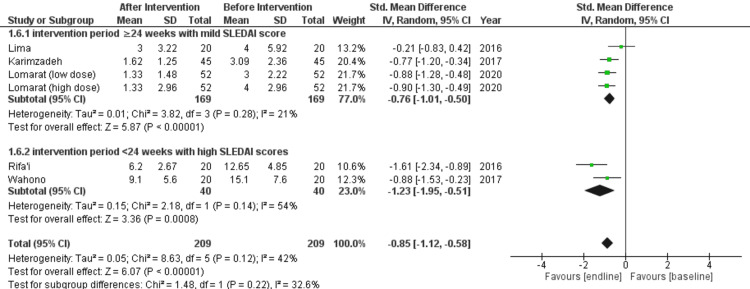
Results for SLEDAI SLEDAI, systemic lupus erythematosus disease activity index.

There was a statistically significant decrease in SLEDAI in the intervention period greater than 24 weeks with mild SLEDAI scores (SMD = -0.76 (-1.01, -0.50); p < 0.00001; I^2 ^= 21%) and in the intervention period less than 24 weeks with high SLEDAI scores (SMD = -1.23 (-1.95, -0.51); p = 0.0008; I^2 ^= 54%). Thus, the overall effect of vitamin D on SLEDAI scores in SLE patients was reduced statistically significantly (SMD = -0.85 (-1.12, -0.58); p < 0.00001; I^2 ^= 42%) (Figure 2).

Effect of Vitamin D on Anti-dsDNA in SLE Patients

**Figure 3:**
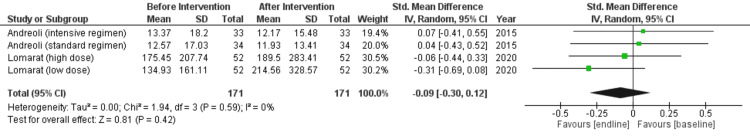
Results for anti-Ds-DNA anti-Ds-DNA: anti-double-stranded DNA.

Two studies were used in this analysis based on their regimen and dosage [19,24]. There were 171 patients in both groups. Pooled analysis showed statistically non-significant decrease in anti-dsDNA (SMD = -0.09 (-0.03, 0.12); p = 0.42; I^2 ^= 0%) (Figure 3).

Effect of Vitamin D on C3 in SLE Patients

**Figure 4:**
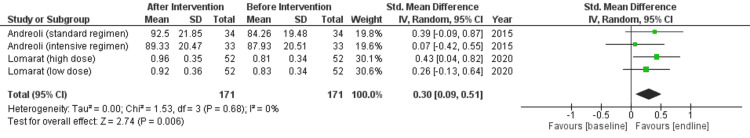
Forest plot showing the effect size of vitamin D's effect on C3

Two studies were used in this analysis based on their regimen and dosage [19,24]. There were 171 patients in both groups. Pooled analysis showed statistically significant increase in C3 (SMD = 0.30 (0.09, 0.51); p = 0.006; I^2 ^= 0%) (Figure 4).

Effect of Vitamin D on C4 in SLE Patients

**Figure 5:**
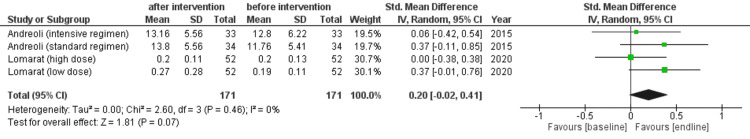
Forest plot showing the effect size of vitamin D's effect on C4

Two studies were used in this analysis based on their regimen and dosage [19,24]. There were 171 patients in both groups. Pooled analysis showed statistically non-significant increase in C4 (SMD = 0.20 (-0.02, 0.41); p = 0.07; I^2 ^= 0%) (Figure 5).

Effect of Vitamin D on Fatigue in SLE Patients

Two studies were used to analyze the effect of vitamin D on fatigue in SLE patients [20,21]. Both groups had 40 patients each. Analysis was done by subgrouping fatigue when performing an exercise, being fatigued easily, fatigue considered a major problem, fatigue affecting duties and responsibilities, fatigue interfering in social life, and Fatigue Severity Score (FSS).

**Figure 6:**
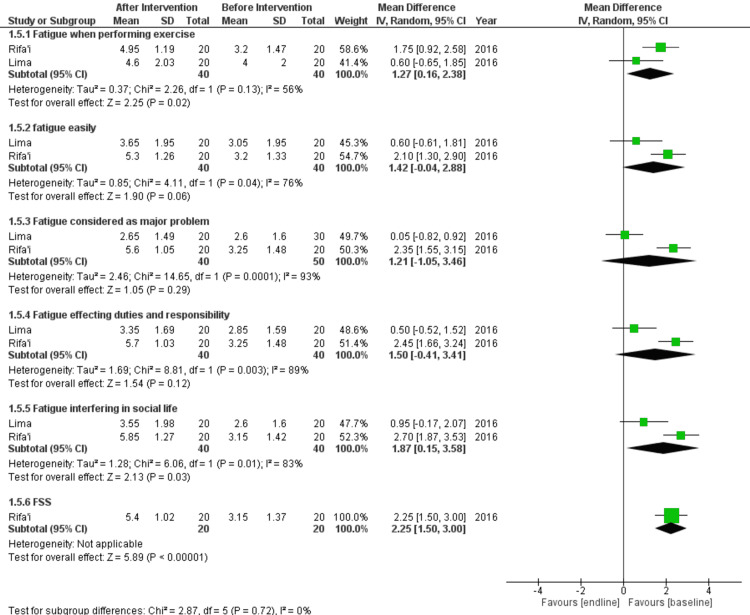
Results for fatigue

There was a statistically significant increase in fatigue when performing an exercise (SMD = -1.27 (-2.38, -0.16); p = 0.02; I^2 ^= 56%), fatigue interfering in social life (SMD = -1.87 (-3.58, -0.15); p = 0.03; I^2 ^= 83%), and FSS (SMD = -2.25 (-3.00, -1.50); p < 0.00001; I^2 ^= not applicable). There was a statistically non-significant increase in being fatigued easily (SMD = -1.40 (-2.86, 0.07); p = 0.06; I^2 ^= 77%), fatigue considered as major problem (SMD = -1.22 (-3.47, 1.04); p = 0.29; I^2 ^=92%), and fatigue affecting duties and responsibilities (SMD = -1.50 (-3.41, 0.41); p = 0.12; I^2 ^= 88%) (Figure 6).

Sensitivity Analysis

The sensitivity analysis was conducted to assess the influence of individual studies on the overall effect. Every study was excluded individually, followed by the generation of pooled SMD for the rest of the studies. After excluding any individual study, no significant change was observed, indicating the results were robust.

Discussion

In this meta-analysis, evidence from six RCTs [19-24] was assessed to evaluate the effectiveness of vitamin D therapy on disease activity, fatigue, and serological markers of SLE patients. Data on SLEDAI scores before and after vitamin D supplementation showed significant improvements. Depending on the duration of vitamin D supplementation, studies were analyzed into two subgroups: intervention for more than 24 weeks and less than 24 weeks. Two studies assessed fatigue using FSS scores and revealed significant improvement in FSS. Serological marker C3 was significantly improved; however, C4 and anti-dsDNA were found insignificant.

We analyzed the data from five RCTs for the effects of vitamin D supplementation on SLEDAI scores. The SLEDAI scores were improved in both patient groups, i.e., patients with mild SLEDAI score treated for >24 weeks and patients with high SLEDAI scores treated for <24 weeks.

Several cross-sectional studies have shown an association between low levels of vitamin D in SLE patients as compared to healthy controls [25,26]. SLE patients are often photosensitive and known to avoid sunlight, which can lead to flares of symptoms like arthralgia and fatigue [27]. Moreover, glucocorticoid is a commonly used drug in SLE. It increases vitamin D hydroxylase expression with early catabolism of vitamin D in the kidney. Islam et al. showed that vitamin D is significantly decreased in SLE patients during summer when compared to the control group and the use of corticosteroids and immunosuppressants was significantly associated with low levels of vitamin D [13].

Previously, in a meta-analysis, Zheng et al. assessed the effect of vitamin D supplementation in SLE patients in comparison to the placebo group. The study showed non-significant relation of vitamins with the disease activity and anti-dsDNA. They found vitamin D supplement was only beneficial in improving serum vitamin D levels and fatigue. However, the assessed results include the data from a retracted study [15].

In individuals genetically predisposed to SLE, there is a loss of tolerance to self-antigens and increased production of autoantibodies like anti-dsDNA [28]. Vitamin D deficiency is expressed with loss of regulatory T cells and an increase in Th1 and Th17 phenotypes [29]. Cells of our immune system like T cells and B cells express vitamin D receptors on their surface and vitamin D deficiency has been linked with an increase in autoreactive B cells [30], with a subsequent increase in autoantibodies like anti-dsDNA. The inhibitory effects of vitamin D on the production of autoantibodies correlate with a decrease in the SLEDAI scores, after a decrease in the inflammatory process of SLE [31].

A retrospective cohort study showed that SLE patients with hypovitaminosis D have significantly low levels of anti-dsDNA [25]. In a clinical trial by Terrier et al., vitamin D supplementation was given to hypovitaminosis D patients, which resulted in an increase in regulatory T cells and a decrease in memory B cells along with anti-dsDNA [29]. Wahono et al. assessed the effects of vitamin D supplementation on anti-dsDNA in hypovitaminosis D SLE patients and found a significant improvement in anti-dsDNA after three and 12 months of vitamin D supplementation as compared to the placebo group, respectively [23]. Zheng et al. found an insignificant association between anti-dsDNA positivity and vitamin D supplementation. The effects of vitamin D supplementation in SLE patients on serum anti-dsDNA levels were insignificant in this analysis [15]. The two RCTs in our analysis reported the data on patients with low baseline SLEDAI scores and most of the patients had normal levels of vitamin D at baseline. Inadequate sample size and inclusion of patients with normal vitamin D levels are a possible risk of bias in these studies.

In SLE patients, vitamin D deficiency is significantly correlated with an increase in nuclear factor kappa B (NF-kB), leading to an increase in tumor necrotic factor-a (TNF-a) [32]. TNF-a is an acute phase reactant that is associated with drowsiness and fatigue in SLE patients [33]. A cohort study assessed the response of two years of vitamin D therapy in SLE on fatigue measured by a visual analog scale [34]. They reported a significant reduction in fatigue after vitamin D therapy. Zheng et al. also found a significant improvement in the overall FSS score in vitamin D supplemented patients [15]. We assessed each component of FSS individually and found a significant improvement in fatigue when performing an exercise and fatigue interfering with social life. Fatigue interfering in social life is a specifically important component of FSS in young adults. Nonetheless, after the intervention, there was no improvement in being fatigued easily and fatigue affecting duties and responsibilities was the major complaint. However, the available data for fatigue and the FSS scale were not strong enough to predict the effects of vitamin D in improving the life of SLE patients.

The complement system is a component of the host’s immune response, activated by either alternate (innate immune system) pathway or a classical pathway (adaptive immune system) [35]. Immune complexes formed in SLE can activate the classical pathway by binding with C1q protein and lead to the splitting of C3 and C4 complement proteins [35]. The complement cascade mediates the activity of phagocytic macrophages by amplifying the opsonization of apoptotic cells through C1q, C4b, and C3b [36]. A deficiency of these complements can result in impaired clearance of autoantigens and an increase in the autoimmune response in SLE patients [36]. A routine assessment of C3 and C4 in SLE patients helps in clinical management, as their levels are decreased in clinically active disease with hypovitaminosis D [35].

A cohort study found a significantly positive correlation between low vitamin D and C4 levels (p = 0.014), and an insignificant correlation with C3 levels (p = 0.191) [25]. This analysis showed a significant improvement in C4 levels, but not in C3 levels. A reason for the results of C3 can be the autoregulation of the classical pathway by C4-binding protein [35,37]. Another reason could be the inclusion of RCTs with low baseline SLEDAI scores, inadequate sample size, and patients having normal levels of vitamin D at baseline.

The statistical result showed that vitamin D significantly improves disease activity. No correlation of vitamin D with anti-dsDNA, fatigue, and complement system was established due to the small availability of data. Along with the lack of data availability, the reason for inconclusive results could be variation in baseline disease activity in patients and different doses of vitamin D. We suggest the use of vitamin D in SLE can improve the disease activity. Longer trials should be conducted on this subject to evaluate the role of VDR and vitamin D supplements in SLE.

Limitations

This study is limited by the following reasons: (a) few studies were available to conduct the analysis; (b) less than 500 patients were included in our study, which might result in a bias; (c) there was a high heterogeneity seen in results of fatigue. These studies were vital for the analysis; however, more studies with the community and random controls should be conducted.

## Conclusions

In this systematic review and meta-analysis, we evaluated the effects of vitamin D supplements on SLE patients. The results of the analysis showed a significant decrease in SLEDAI score, irrespective of the duration of treatment and severity of the disease. The fatigue score and C3 were significantly improved. However, vitamin D showed no role in improving anti-dsDNA and C4. Further research should be conducted to evaluate the role of vitamin D on disease activity and autoimmunity.

## Figures and Tables

**Figure 7:**
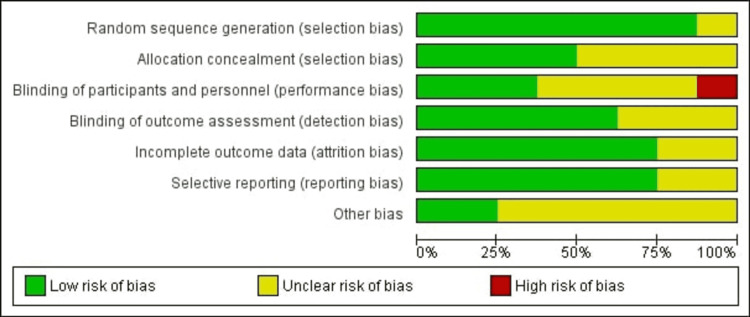
Quality assessment graph

**Figure 8:**
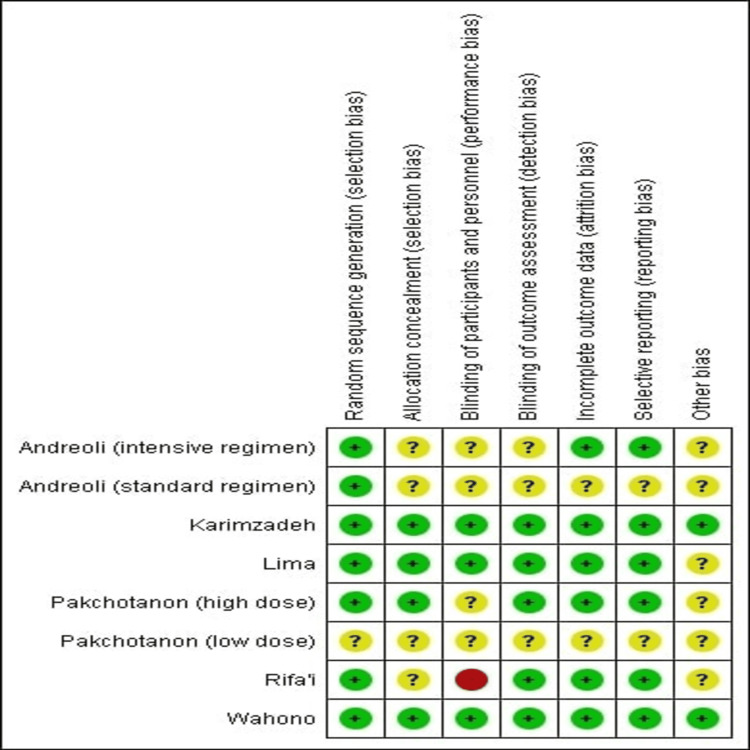
Quality assessment chart

## Appendices

**Table 3:** Quality assessment of the selected articles using Cochrane Collaboration’s tool

Study	Adequate sequence generation	Allocation concealment	Blinding of participants and personnel	Blinding of outcome assessment	Incomplete outcome data	Selective outcome reporting	Free of other bias	Net risk of bias
Andreoli et al. [19]	Low risk	Unclear risk	Unclear risk	Unclear risk	Low risk	Low risk	Unclear risk	Moderate risk
Lima et al. [20]	Low risk	Low risk	Low risk	Low risk	Low risk	Low risk	Unclear risk	Low risk
Rifa’i et al. [21]	Low risk	Unclear risk	High risk	Low risk	Low risk	Low risk	Unclear risk	Low risk
Karimzadeh et al. [22]	Low risk	Low risk	Low risk	Low risk	Low risk	Low risk	Low risk	Low risk
Wahono et al. [23]	Low risk	Low risk	Low risk	Low risk	Low risk	Low risk	Low risk	Low risk
Pakchotanon et al. [24]	Low risk	Low risk	Unclear risk	Low risk	Low risk	Low risk	Unclear risk	Low risk
